# Expression of inflammasome proteins and inflammasome activation occurs in human, but not in murine keratinocytes

**DOI:** 10.1038/s41419-017-0009-4

**Published:** 2018-01-18

**Authors:** Jennifer Sand, Eric Haertel, Thomas Biedermann, Emmanuel Contassot, Ernst Reichmann, Lars E. French, Sabine Werner, Hans-Dietmar Beer

**Affiliations:** 1 Department of Dermatology, University Hospital Zurich, Gloriastrasse 31, F floor, Zurich, 8091 Switzerland; 2 Department of Biology, Institute for Molecular Health Sciences, ETH Zürich, Otto-Stern-Weg 7, Zurich, 8093 Switzerland; 3 Department of Surgery, Tissue Biology Research Unit, University Children’s Hospital Zurich, August-Forel-Strasse 7, Zurich, 8008 Switzerland; 4 Faculty of Medicine, University of Zurich, Zurich, Switzerland

## Abstract

Inflammasomes are multimeric protein complexes that assemble upon sensing of a variety of stress factors. Their formation results in caspase-1-mediated activation and secretion of the pro-inflammatory cytokines pro-interleukin(IL)-1β and -18, which induce an inflammatory response. Inflammation is supported by a lytic form of cell death, termed pyroptosis. Innate immune cells, such as macrophages or dendritic cells, express and activate inflammasomes. However, it has also been demonstrated that human primary keratinocytes activate different types of inflammasomes in vitro, for example, upon UVB irradiation or viral infection. Keratinocytes are the main cell type of the epidermis, the outermost layer of the body, and form a protective barrier consisting of a stratified multi-layered epithelium. In human, gain-of-function mutations of the *NLRP1* gene cause syndromes mediated by inflammasome activation in keratinocytes that are characterised by skin inflammation and skin cancer susceptibility. Here we demonstrate that murine keratinocytes do not activate inflammasomes in response to stimuli, which induce IL-1β and -18 secretion by human keratinocytes. Whereas murine keratinocytes produced caspase-1 and proIL-18, expression of the inflammasome proteins Nlrp1, Nlrp3, Aim2, Asc, and proIL-1β was, compared to human keratinocytes or murine dendritic cells, very low or even undetectable. Priming of murine keratinocytes with cytokines commonly used for induction of proIL-1β and inflammasome protein expression did not rescue inflammasome activation. Nevertheless, UVB-induced inflammation and neutrophil recruitment in murine skin was dependent on IL-1β and caspase-1. However, also under these conditions, we did not detect expression of proIL-1β by keratinocytes in murine skin, but by immune cells. These results demonstrate a higher immunological competence of human compared to murine keratinocytes, which is reflected by stress-induced IL-1β secretion that is mediated by inflammasomes. Therefore, keratinocytes in human skin can exert immune functions, which are carried out by professional immune cells in murine skin.

## Introduction

The skin is the outermost layer of the body and provides an efficient barrier for protection against pathogens and different types of mechanical, physical or chemical insults^1^. The two main compartments of the skin are the dermis, a connective tissue that provides support and elasticity, and the epidermis, which forms a strong barrier by a well-structured organisation of keratinocytes in different cell layers. The latter cell type synthesises the major structural components of the epidermal barrier by a tightly controlled process termed terminal differentiation, resulting in the sealing of the epidermis by an envelope of flattened and transcriptionally inactive corneocytes that are embedded in a lipid bilayer^2,3^. Cells of the innate and adaptive branch of the immune system further regulate tissue homoeostasis through recognition and elimination of harmful pathogens and respond to stress factors and injuries. Upon induction of inflammation, repair processes are initiated in order to re-establish homoeostasis^4^. Within the skin, resident dendritic cells, macrophages, neutrophils, mast cells and T cells, but also keratinocytes act immediately as innate immune sensors, thereby promoting additional immune cell responses and inflammation^5,6^.

Ultraviolet (UV) radiation from sunlight represents a major environmental threat and stress factor for the skin. Excessive exposure to UV can directly damage DNA of epidermal cells, but also induces the formation of reactive oxygen species (ROS), which can cause additional DNA modifications^7^. This initiates either cell survival pathways upon DNA repair or programmed cell death of irreversibly damaged keratinocytes^8^. In addition, excessive exposure to UVB induces inflammation of the skin, commonly termed sunburn. At the cellular level, this is characterised by the recruitment  of neutrophils, while at the molecular level, activation of several stress pathways occurs, including the nuclear factor (NF)-κB pathway^9^. In addition, inflammasomes are believed to contribute to UVB-induced inflammation^10,11^.

Inflammasomes are cytosolic protein complexes composed of a nucleotide-binding/leucine-rich repeat (NLR) or absent in melanoma (AIM2-like) receptor (ALR), the adaptor protein apoptosis-associated speck-like protein containing a CARD (ASC) and the protease caspase-1. Sensing of diverse stimuli by different inflammasome sensors like NLRP1, NLRP3 or AIM2 results in assembly of the sensor with ASC and caspase-1, leading to caspase-1-dependent maturation and secretion of the pro-inflammatory cytokines proIL-1β and proIL-18, along with other proteins^12^. Inflammasomes are well characterised in immune cells of myeloid origin, but their components are also expressed by some non-professional immune cells. Furthermore, different inflammasomes can have distinct tissue- or cell type-specific functions^12,13^.

*In vitro*, human keratinocytes mediate inflammasome-dependent secretion of mature IL-1β and IL-18 in response to UVB irradiation, viral infections or transfection with DNA^10,14–16^. Furthermore, UVB exposure induces caspase-1-dependent apoptosis, which might antagonise skin cancer development by eradication of damaged keratinocytes^17^. Most importantly, gain-of-function mutations in the *NLRP1* gene cause syndromes in human that are characterised by skin inflammation and skin cancer susceptibility mediated by keratinocytes^18^.

Although mice are widely used to study skin inflammation and mechanisms underlying inflammatory skin diseases, the response of murine keratinocytes* in vitro* to UVB is only partially characterised. We previously showed that recruitment of neutrophils upon UVB irradiation of murine skin is dependent on caspase-1^10^, suggesting a similar function of this protein in the sunburn reaction of murine and human skin. However, in contrast to human keratinocytes, caspase-1 expression is dispensable for UVB-induced apoptosis in murine keratinocytes *in vitro* and in vivo^17^. Human keratinocytes constitutively express inflammasome components without the need of a priming step^10,18^, whereas expression of Nlrp3 could not be detected in their murine counterparts^19^. Additionally, expression of proIL-1β by murine keratinocytes is a matter of debate^20–23^. In a study of chemically induced skin carcinogenesis in mice, the authors suggested that IL-1β is mainly secreted by infiltrating myeloid cells rather than by keratinocytes^24^. Murine keratinocytes can secrete IL-1α upon nanoparticle stimulation independently of Asc, whereas IL-1β secretion could not be demonstrated under these conditions^25^. In contrast, a role of caspase-1-dependent IL-1 secretion by keratinocytes in tissue repair responses upon UVR damage in murine skin has been suggested^22^. In summary, it remains to be determined, whether murine keratinocytes can activate inflammasome complexes like their human counterparts.

Here we provide evidence for a fundamentally different response to UVB irradiation between keratinocytes of human vs. murine skin. In particular, our results suggest that—in contrast to human skin—the response to UVB, and eventually “sunburn”, in mice is not dependent on keratinocytes but most likely mediated by professional immune cells.

## Materials and methods

### Mice

C57BL/6 mice (*wt*) were purchased from Janvier Laboratories (Le Genest-Saint-Isle, France). Initially, *Caspase-1*^−*/−*^ mice were kindly provided by Dr C. Grimm (University Hospital Zurich, Zurich, Switzerland). They were backcrossed to the C57BL/6 genetic background for five generations and bred in house for all experiments. *Lang-DTREGFP*^26^ and *IL-1β*^*−/−*^^27^ mice were described. Mice were housed in individually ventilated cages under specific pathogen-free conditions. All experiments were approved by the local veterinary authorities.

### UVB irradiation

For UVB irradiation of back skin, mice were anaesthetised by i.p. injection of a mix of ketamine (0.06 mg/g body weight) and xylazine (0.003 mg/g body weight). Afterwards, mice were shaved on the back with an electric animal shaver (Favorita II, Aesculap AG, Suhl, Germany) and the eyes were covered with vitamin A eye cream to prevent dryness (Bausch&Lomp Swiss AG, Zug, Switzerland). Mice were placed below a UVB light source (UV802L, Waldmann, Villingen-Schwenningen, Germany) at a distance of 10 cm and irradiated for 24.7 min, equal to a dose of 300 mJ/cm^2^. The heads were covered with aluminium foil.

### Cell culture

Isolation and culture of human primary foreskin keratinocytes (HFKs) have been previously described^10,28,29^. Briefly, HFKs were passaged in keratinocyte-SFM (K-SFM, Gibco, Paisley, Scotland), supplemented with epidermal growth factor and bovine pituitary extract (Gibco, Paisley, Scotland). Cells were seeded for experiments after passage 3. For siRNA-mediated knock-down experiments, the cells were seeded in 6-well plates at a density of 1 × 10^5^ cells/well. The day after seeding, cells were transfected with siRNA oligonucleotides (10 nM, Sigma (Munich, Germany)/Microsynth (Balgach, Switzerland)) using 2 µl INTERFERin (Polyplus, Illkirch, France) as a transfection reagent and left for 48 h. Preparation of human organotypic skin was performed as described^30^.

### Knock-down with siRNA in human primary keratinocytes

Human primary keratinocytes were transfected with siRNAs (10 nM) targeting the inflammasome proteins caspase-1, caspase-5, NLRP1, ASC and a control siRNA (scrambled) using INTERFERin (Polyplus, Illkrich, France). siRNAs were purchased from Microsynth (Balgach, Switzerland) or Sigma (Munich, Germany). The following siRNAs were used: scrambled siRNA: 5′-UUCUCCGAACGUGUCACGU-3′; caspase-1 siRNA: 5′-GGCAGAGAUUUAUCCAAUA-3′; caspase-5 siRNA: 5′-GUGGCUGG CAAACAUCUA-3′; NLRP1 siRNA: 5′-GCUUCUGCUCGCCAAUAAAA-3′; ASC siRNA: 5′-GCUUCUACCUGGAGACCUA-3′.

### Real-time PCR

Levels of mRNA of inflammasome proteins were determined with quantitative real-time PCR (qRT-PCR)  using the LightCycler 480 SYBR Green Master (Roche, Rotkreuz, Switzerland) and specific primers were purchased from Microsynth. The following primers were used for murine genes: Nlrp3 forward 5′-ATTACCCGCCCGAGAAAGG-3′; Nlrp3 reverse 5′-TCGCAGCAAAGATCCACACAG-3′; Aim2 forward 5′-GTCACCAGTTCCTCAGTTGTG-3′; Aim2 reverse 5′-CACCTCCATTGTCCCTGTTTTAT-3′; Nlrp1 forward 5′-ATGTGGACCCAACCTTCAAA-3′; Nlrp1 reverse 5′-GTACGTGCTCCTGGAAAGGT-3′; IL-1β forward 5′-GCAACTGTTCCTGAACTCAACT-3′; IL-1β reverse 5′-ATCTTTTGGGGTCCGTCAACT-3′; Asc forward 5′-CTTGTCAGGGGATGAACTCAAAA-3′; Asc reverse 5′-GCCATACGACTCCAGATAGTAGC-3′; caspase-1 forward 5′-ACAAGGCACGGGACCTATG-3′; caspase-1 reverse 5′-TCCCAGTCAGTCCTGGAAATG-3′; IL-1α forward 5′-GCACCTTACACCTACCAGAGT-3′; IL-1α reverse 5′-AAACTTCTGCCTGACGAGCTT-3′; IL-18 forward 5′-GACTCTTGCGTCAACTTCAAGG-3′; IL-18 5′-CAGGCTGTCTTTTGTCAACGA-3′. The following primers were used for human genes: NLRP3 forward 5′-GCAAAAAGAGATGAGCCGAAG T-3′; NLRP3 reverse 5′-GCTGTCTTCCTGGCATATCACA-3′; AIM2 forward 5′-CAGAAATGATGTCGCAAAGCA-3′; AIM2 reverse 5′-TCAGTACCATAACTGGCAAACAG-3′; NLRP1 forward 5′-CAGGCAGCACAGATCAACAT-3′; NLRP1 reverse 5′-GTGACCTTGAGGACGGAGAA-3′; IL-1β forward 5′-CACGATGCACCTGTACGATCA-3′; IL-1β reverse 5′-GTTGCTCCATATCCTGTCCCT-3′; ASC forward 5′-CGCGAGGGTCACAAACGT-3′; ASC reverse 5′-TGCTCATCCGTCAGGACCTT-3′; CASPASE-1 forward 5′-TCCCTAGAAGAAGCTCAAAGGATATG-3′; caspase-1 reverse 5′-CGTGTGCGGCTTGACTTG-3′; IL-1α forward 5′-ATCATGTAAGCTATGGCCCACT-3′; IL-1α reverse 5′-CTTCCCGTTGGTTGCTACTAC-3′; IL-18 forward 5′-GCTGCTGAACCAGTAGAAGAC-3′; IL-18 reverse 5′-CCGATTTCCTTGGTCAATGAAGA-3′; IL-1RA forward 5′-GGAATCCATGGAGGGAAGAT-3′; IL-1RA reverse 5′-TCTCGCTCAGGTCAGTGATG-3′.

### Dissociation of skin tissue for flow cytometric analysis

Up to half the skin from the back of a mouse was collected and stored in RPMI 1640/HEPES medium on ice. After weighing, amounts of tissue were matched and intensely cut into small pieces using surgical scissors. The resulting mash was transferred to 15 ml conical tubes containing Mg^2+^/Ca^2+^-free 1× PBS followed by addition of EGTA (10 mM final concentration). Tubes were incubated at 37 °C with continuous shaking (65 rpm) for 20 min. Tissue was pelleted by centrifugation and washed twice with PBS. Samples were resuspended in 4 ml RPMI 1640/HEPES medium and pre-digested by addition of Liberase TL (1.3 WU/ml final; Roche, Rotkreuz, Switzerland) followed by incubation at 37 °C and 65 rpm for 60 min. Pre-digested tissue was loosened by vigorous manual shaking and medium containing Dispase II (1 KU/ml final; Gibco, Paisley, UK), bovine DNase I (0.2 mg/ml final; Sigma, Munich, Germany) and MgCl_2_ (7.5 mM final concentration) were added. Samples were incubated at 37 °C and 80 rpm for 15 min, followed by incubation at room temperature and 80 rpm for additional 15 min. The cell suspension was diluted with 1× PBS and passed through a 30 µm cell strainer (CellTrics, Sysmex, Horgen, Switzerland). Cells were pelleted by centrifugation, transferred to a 96-well V-bottom plate (Sarstedt, Nürnbrecht, Germany) and treated for cytokine restimulation or directly labelled for flow cytometric analysis.

### Flow cytometric analysis

Cells were processed in 96-well V-bottom plates (Sarstedt, Nuembrecht, Germany) and all staining and fixation steps were performed at 4 °C in the dark. Cells intended for staining of cytokines were restimulated by incubation in RPMI 1640/HEPES containing CpG (20 nM final), FK565 (600 ng/ml final), flagellin (20 ng/ml final), lipoteichic acid (200 ng/ml final), LPS (20 nM final), Poly:IC (100 ng/ml final) and R837 (1 µg/ml final) at 37 °C and 5% CO_2_ for 1 h, followed by addition of brefeldin-A (BFA, 5 µg/ml final) and monensin (5 µg/ml final) and incubation for additional 3 h at 37 °C. Following restimulation, cells were washed with 1× PBS and stained in 1× PBS containing Zombie Red fixable viability dye and fluorochrome-conjugated antigen-specific antibodies for 30 min. To minimise non-specific binding, Fc receptor block (anti-mouse CD16/CD32) was included. Following cell surface staining (CSS), cells were washed with flow buffer (1× PBS, 2% FBS, 5 mM EGTA, 0.09% NaOAc), fixed for 1 h (FoxP3 staining buffer set, eBioscience, San Diego, CA, USA) and washed once with 1× permeabilisation buffer (Perm buffer, eBioscience). For intracellular staining (ICS), antibodies were diluted in 1× Perm buffer and cells were stained overnight. Cells were then washed with Perm buffer, resuspended in flow buffer, and stored at 4 °C in the dark until acquisition.

Stained cells were analysed using a BD LSRII Fortessa equipped with FACSDiva software (Version 6, BD Pharmingen, San Diego, CA, USA). Compensation of fluorescence emission was performed using compensation beads (BD Biosciences). Samples were acquired using Fortessa’s HTS plate reader option at an event rate below 20,000 events/s. Staining and gating controls included isotype control and fluorescence minus one (FMO) samples. Data analysis was performed using FlowJo software (Version X, Tree Star Inc., Ashland, OR, USA).

### Statistical analysis

All data were statistically analysed using GraphPad Prism 6.0 and 7.02 software (GrapPad Software, Inc., La Jolla, CA, USA). For the analysis of two groups, two-tailed unpaired *t*-test was performed. Comparison of three or more groups was performed using one-way ANNOVA, either with Dunnett’s correction if all groups were compared to one control group, or with Sidak’s or Tukey’s correction, if several groups were compared to other groups. *P* values below 0.05 were considered significantly different (^****^p < 0.0001, ^***^p < 0.001, ^**^p < 0.01, ^*^p < 0.05). All data are displayed as mean +/-standard deviation (SD).

## Results

### UVB irradiation induces secretion of mature IL-1β by human keratinocytes

**Fig. 1:**
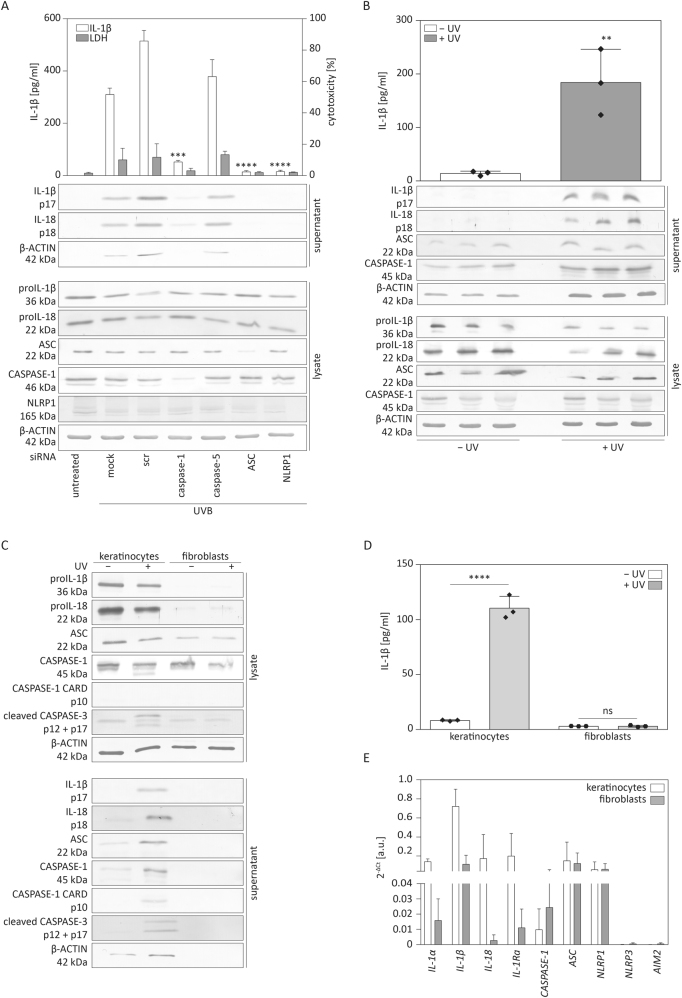
**Human keratinocytes secrete IL-1β and IL-18 upon UVB irradiation** **a** Human primary keratinocytes were transfected with different siRNAs as indicated or scrambled (scr) siRNA as control. Two days later, they were irradiated with UVB (87.5 mJ/cm^2^). Supernatant and cell lysates were harvested after 5 h and analysed for IL-1β in the supernatant by ELISA or by western blot for expression and secretion of the indicated proteins. Cytotoxicity was determined using the lactate dehydrogenase (LDH) assay. **b** Human organotypic skin cultures were exposed to UVB (87.5 mJ/cm^2^) and cells and supernatants were harvested after 24 h. Western blot analysis to detect secretion and expression of the indicated proteins and ELISA to detect secretion of IL-1β were performed. **c**, **d** Subconfluent primary keratinocytes and fibroblasts grown in 2D monoculture were exposed to UVB (87.5 mJ/cm^2^). Supernatants and cells were harvested after 5 h and analysed by western blot (**c**) for expression and secretion of the indicated proteins and for IL-1β in the supernatant by ELISA (**d**). **e** Subconfluent keratinocytes and fibroblasts grown in 2D monoculture were harvested for RNA isolation. mRNA levels of the indicated genes were determined by qRT-PCR relative to the housekeeping gene *RPL27*. Error bars represent mean ± SD of a representative experiment performed in triplicates. **a** One-way ANOVA with Dunnett’s multiple comparisons test comparing all values to scr. **b** Error bars represent mean ± SD of three different cultures. Unpaired *t*-test was performed to compare irradiated to non-irradiated cultures. **c**, **d** Unpaired *t*-test comparing non-irradiated vs. irradiated keratinocytes or fibroblasts (****p <0.0001, ***p < 0.001, **p < 0.01). scr scrambled, LDH lactate dehydrogenase, kDa kilo Dalton, IL interleukin, ns not significant, a.u. arbitrary units

UVB irradiation is a well-established inflammasome activator that leads to maturation and secretion of the pro-inflammatory cytokines proIL-1β and proIL-18 by human primary keratinocytes^10,29,31^. To test for dependency of cytokine secretion on expression of inflammasome proteins, we transfected human primary keratinocytes with different siRNAs targeting mRNAs of inflammasome-related and -unrelated genes and subsequently irradiated the cells with UVB as previously described^10^. Upon transfection of cells with scrambled siRNA or siRNA against caspase-5 we observed maturation and secretion of high levels of IL-1β and -18, 5 h after irradiation (Fig. 1a). However, knock-down of the inflammasome proteins caspase-1, ASC or NLRP1 drastically reduced the secretion of IL-1β and IL-18 (Fig. 1a). These results confirm that UVB irradiation induces activation of the NLRP1 inflammasome in human primary keratinocytes, which results in secretion of large amounts of IL-1β and IL-18.

### Keratinocytes in human organotypic skin cultures secrete IL-1β in response to UVB

To further determine the physiological relevance of inflammasome activation in human keratinocytes, we subjected human organotypic skin cultures that resemble human skin and can be clinically used as skin substitutes^30^ to UVB irradiation. These cultures consist of a collagen layer with embedded human primary fibroblasts and an epidermis-like structure on top, with several layers of keratinocytes at different stages of differentiation. Like human primary keratinocytes in 2D monoculture, skin substitutes secreted mature IL-1β and IL-18 in a UVB-dependent manner (Fig. 1b). As these skin equivalents are composed of keratinocytes and fibroblasts, we analysed whether fibroblasts can secrete IL-1β upon UVB irradiation. Therefore, we subjected fibroblasts and keratinocytes separately to UVB irradiation. Fibroblasts did not secrete mature IL-1β or IL-18 as determined by western blot (Fig. 1c) and ELISA (Fig. 1d). Furthermore, mRNA levels of the cytokines IL-1α, IL-1β and IL-18 were much higher in keratinocytes than in fibroblasts grown in 2D monoculture (Fig. 1e). Hence, human primary keratinocytes secrete IL-1β and IL-18 not only upon UVB irradiation in monoculture but also under more physiological conditions in a three-dimensional skin model.

### Inflammasomes cannot be activated in murine keratinocytes

**Fig. 2:**
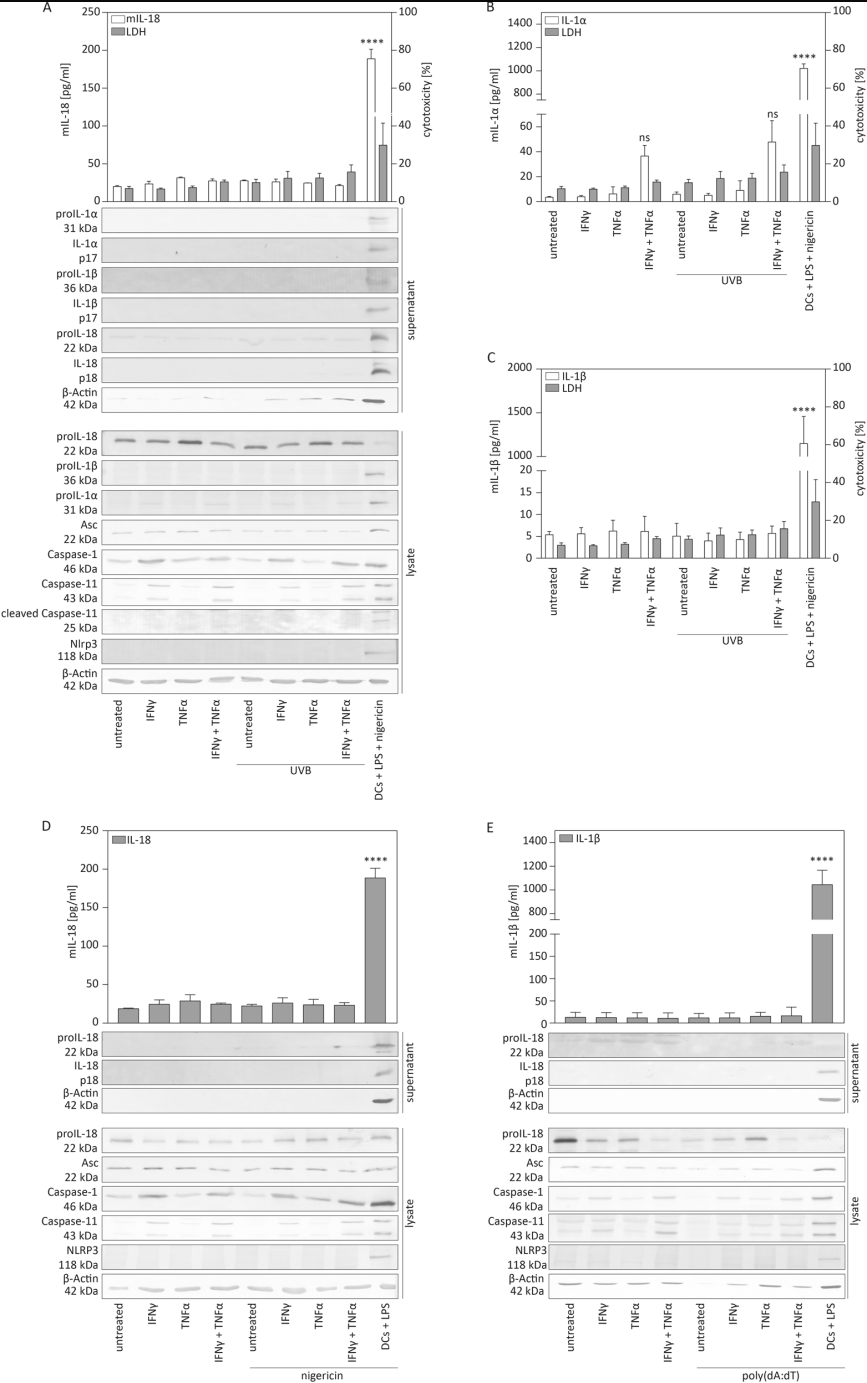
**Established inflammasome activators do not induce secretion of IL-1β and IL-18 by murine keratinocytes** **a**–**e** Murine primary keratinocytes were left untreated or primed with IFNγ and/or TNFα for 16 h and subsequently irradiated with UVB (87.5 mJ/cm^2^). **a** Supernatant and cells were harvested after 5 h and analysed for IL-18 in the supernatant by ELISA, for cytotoxicity by LDH assay and by western blot for secretion and expression of the indicated proteins. Murine bone marrow-derived dendritic cells, primed with LPS for 16 h and treated with nigericin (5 μM) for 6 h, served as control (last bars or lane on the right side). **b** Supernatant and cells were harvested after 5 h and analysed for IL-1α secretion by ELISA and for cytotoxicity by LDH assay. **c** Supernatant and cells were harvested after 5 h, analysed for IL-1β secretion by ELISA and for cytotoxicity by LDH assay. **d** Murine keratinocytes were stimulated with nigericin (5 μM). After 6 h, supernatant and cells were harvested and analysed for IL-18 in the supernatant by ELISA or by western blot for secretion or expression of the indicated proteins. Murine bone marrow-derived dendritic cells (DCs), primed with LPS, served as a control (last bars or lane on the right side). **e** Murine primary keratinocytes were transfected with poly(dA:dT) (4 μg/ml). After 20 h, supernatants and cells were harvested and analysed for IL-1β in the supernatant by ELISA or by western blot for expression or secretion of the indicated proteins. For ELISA, DCs, primed with LPS for 16 h, treated with nigericin (5 μM) and harvested after 6 h served as control (last bar on the right side). Error bars represent mean ± SD of three independent experiments. One-way ANOVA with Dunnett’s multiple comparisons test comparing all values to those of untreated cells was performed (****p < 0.0001). IFNγ interferon-γ, TNFα tumour necrosis factor α, LPS lipopolysaccharide, LDH lactate dehydrogenase, kDa kilo Dalton, IL interleukin, DCs dendritic cells

Next, we addressed whether UVB irradiation induces pro-inflammatory cytokine secretion by murine keratinocytes. Inflammasome activation is established for murine immune cells, such as dendritic cells (DCs). These cells require additional priming to induce optimal transcription of genes encoding inflammasome proteins and pro-inflammatory cytokines^32^. In contrast, human keratinocytes constitutively express inflammasome components^10,18^. We treated murine primary keratinocytes with TNFα and/or IFNγ or left them unprimed, and subjected them to UVB irradiation. Murine bone marrow-derived dendritic cells (BMDCs), primed with LPS and stimulated with the NLRP3 inflammasome activator nigericin, served as control. The overall amount of detected IL-18 in the supernatant of keratinocytes was very low compared to BMDCs and we did not detect IL-18 upregulation upon UVB irradiation compared to non-irradiated control cells (Fig. 2a). Similarly, neither secretion of IL-1α nor of IL-1β was significantly increased upon UVB irradiation, whereas LPS/nigericin stimulation of BMDCs resulted in strong pro-inflammatory cytokine secretion (Fig. 2b, c). Western blotting revealed that, apart from proIL-18, expression of inflammasome and inflammasome-related proteins was much lower or even undetectable in murine keratinocytes compared to BMDCs (Fig. 2a). Priming by IFNγ increased the expression of proIL-1α, caspase-1 and caspase-11 as observed by analysis of cell lysates. However, this did not result in the release of considerable levels of IL-1α, IL-1β or IL-18 by murine keratinocytes upon UVB irradiation (Fig. 2a, b, c).

Human keratinocytes also secrete pro-inflammatory cytokines in an inflammasome-dependent manner in response to other stimuli, such as nigericin or poly(dA:dT)^15^. Therefore, we treated murine primary keratinocytes with nigericin (Fig. 2d) or transfected them with poly(dA:dT), an established inducer of AIM2 inflammasome activation (Fig. 2e)^15,33^. However, none of these stimuli resulted in the release of detectable levels of IL-1β or IL-18, whereas BMDCs secreted large amounts of these pro-inflammatory cytokines (Fig. 2d, e). These results demonstrate that murine keratinocytes do not activate inflammasomes, at least under conditions that result in cytokine secretion by human keratinocytes or murine BMDCs.

### Genes encoding inflammasome components are only weakly expressed in murine keratinocytes

**Fig. 3:**
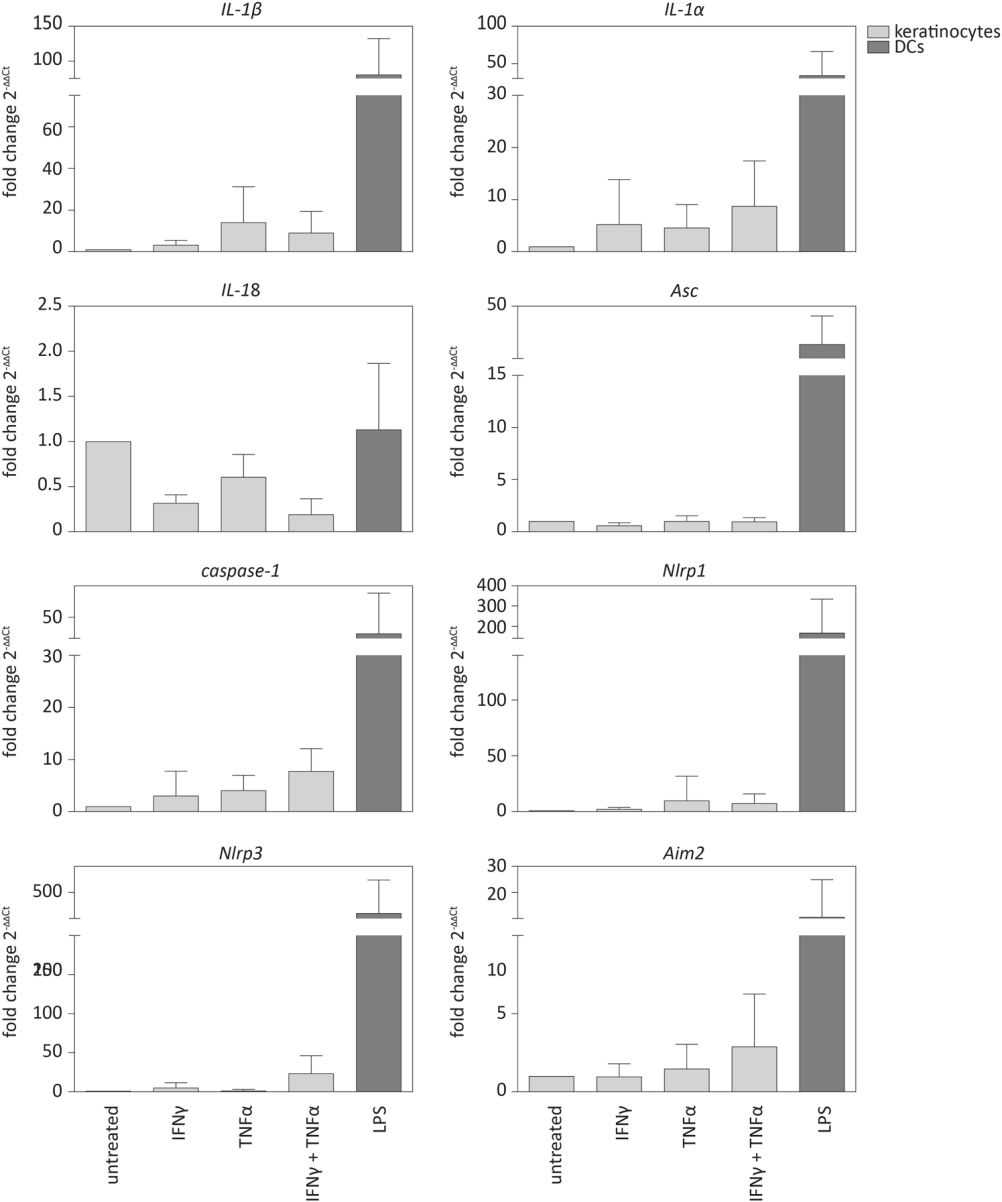
**mRNA levels of genes encoding inflammasome proteins and of IL-1α, IL-1β or IL-18 in murine keratinocytes** Murine immortalised keratinocytes and bone marrow-derived dendritic cells (BMDCs) as positive control were primed with either IFNγ and/or TNFα or with LPS for 16 h. mRNA levels of the indicated genes were determined by qRT-PCR relative to *Rpl27* levels. Error bars represent mean ± SD of *n* = 4 different experiments. One-way ANOVA with Dunnett’s multiple comparisons test comparing all values to those of untreated cells was performed (****p < 0.0001, ***p < 0.001, **p < 0.01, *p < 0.05). IFNγ interferon-γ, TNFα tumour necrosis factor α, LPS lipopolysaccharide, DCs dendritic cells, a.u. arbitrary units, IL interleukin

As we did not detect proIL-1β protein expression in murine keratinocytes and much lower amounts of inflammasome proteins and of proIL-1α compared to BMDCs (Fig. 2a), we analysed whether this was also reflected at the mRNA level in immortalised murine keratinocytes, stimulated with IFNγ and/or TNFα (Fig. 3). Priming resulted in a mild, albeit not statistically significant, increase in the mRNA levels of *IL-1α*, while expression of this cytokine in LPS-treated BMDCs was much higher. As previously described, *Nlrp3* mRNA levels were very low in murine keratinocytes compared to BMDCs^19^, and similar findings were obtained for *Asc*, *caspase-1*, *Nlrp1* and *Aim2*. Most importantly and consistent with previous data^20,21^, while proIL-1β mRNA levels were high in BMDCs, mRNA expression was very low in murine keratinocytes, even upon priming (Fig. 3). Additionally, proIL-1β protein expression was not detectable by western blot (Fig. 2a, d, e). Taken together, these results demonstrate low or undetectable mRNA and protein levels of most inflammasome components in murine keratinocytes, even upon priming. These findings suggest a mechanistic explanation of the lack of inflammasome activation and IL-1β secretion by these cells.

### Recruitment of neutrophils upon UVB irradiation of murine skin is dependent on caspase-1

**Fig. 4:**
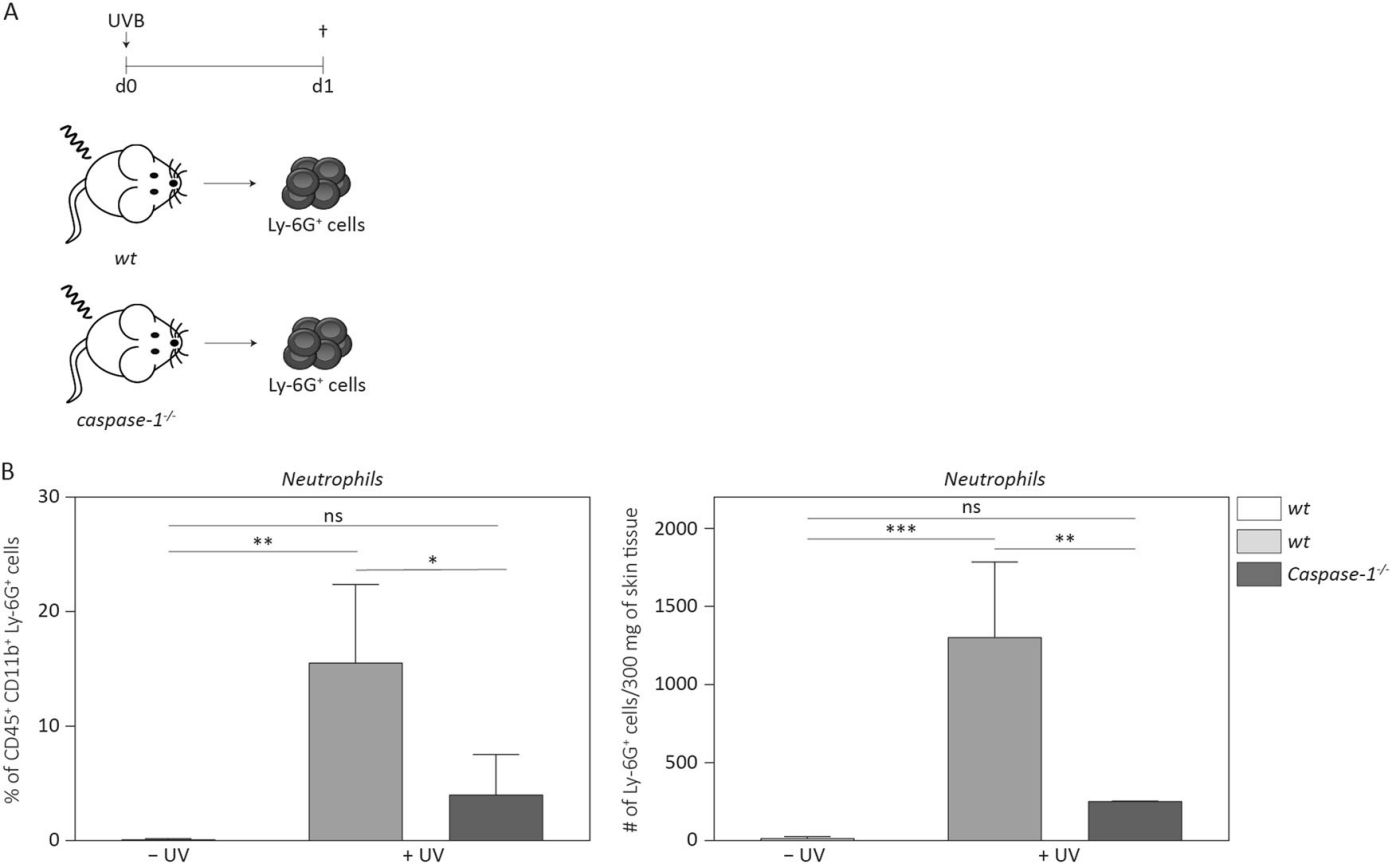
**Neutrophil recruitment upon UVB irradiation is dependent on caspase-1** **a** Wild-type (*wt*) and *Caspase-1*^−*/−*^ mice were shaved on the back and irradiated with UVB (300 mJ/cm^2^). **b** Twenty-four hours after irradiation, back skin was analysed for the percentage of CD45^+^ CD11b^+^ Ly-6G^+^ neutrophils among all cells and for the total number of neutrophils in 300 mg skin tissue. Error bars represent mean ± SD of a representative experiment with *n* = 3 animals per group. One-way ANOVA with Tukey’s multiple comparison was performed (***p < 0.001, **p < 0.01, *p < 0.05). wt wild-type, ns not significant

The skin is drained by blood and lymphatic vessels that allow the trafficking and recruitment of immune cells in response to stress. To determine whether initiation of sunburn in mice is dependent on caspase-1, we irradiated shaved *Caspase-1*^*−/−*^ and wild-type (*wt*) mice with a single dose of UVB and quantified the recruitment of neutrophils as a readout for inflammation, since these cells are typically the first immune cells to accumulate in acutely inflamed skin^34^. Twenty-four hours after irradiation, reddening and swelling of the skin of irradiated mice was already macroscopically visible, and this was accompanied by a strong influx of Ly-6G-positive neutrophils, as determined by flow cytometry (Fig. S1). Importantly, however, both, the percentage and number of CD45^+^ CD11b^+^ Ly-6G^+^ neutrophils were reduced in caspase-1-deficient animals compared to wild-type mice (Fig. 4a, b), consistent with previous results obtained by immunohistochemistry^10,35^.

### Recruitment of neutrophils into murine skin upon UVB irradiation is dependent on IL-1β

**Fig. 5:**
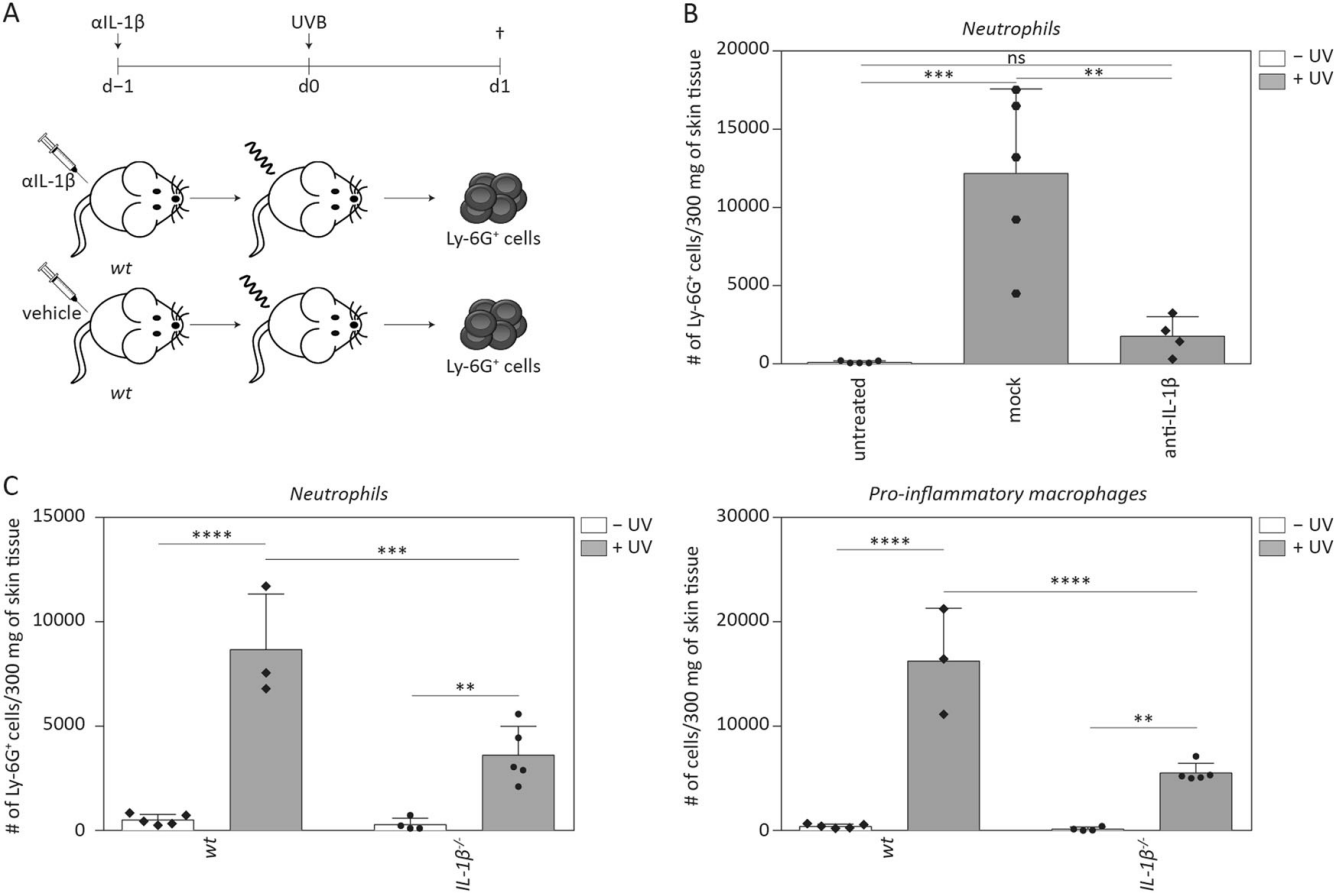
**Neutrophil recruitment upon UVB irradiation is dependent on IL-1β** **a** Experimental setup for IL-1β depletion. Mice were injected with an anti-IL-1β depleting antibody (200 μg/mouse, isotype IgG2aκ) 24 h before irradiation with 300 mJ/cm^2^ UVB. **b** The number of CD45^+^ CD11b^+^ Ly-6G^+^ cells in 300 mg of skin tissue was determined using flow cytometry. **c** Quantification of CD45^+^ CD11b^+^ Ly-6G^+^ neutrophils and of CD45^+^ CD64^hi^ Ly-6C^hi^ pro-inflammatory macrophages per 300 mg of skin tissue in response to UVB irradiation of *IL-1β*^*−/−*^ and *wt* animals. Error bars represent mean ± SD of a representative experiment with *n* = 5 animals/group. One-way ANOVA with Sidak’s multiple comparison was performed (****p < 0.0001, ***p < 0.001, **p < 0.01). ns not significant, d day, IL interleukin

Next, we addressed whether IL-1β expression has an impact on induction of sunburn upon UVB irradiation of murine skin. For this purpose, we injected a neutralising anti-IL-1β antibody into wild-type mice and subjected these animals to UVB irradiation (Fig. 5a). The recruitment of neutrophils upon UVB irradiation was strongly reduced in mice treated with anti-IL-1β antibody compared to control animals (Fig. 5b). To verify the important role of IL-1β in UV-induced inflammation, we irradiated *IL-1β*^*−/−*^ mice and, indeed, detected a reduced number of neutrophils in UVB-irradiated *IL-1β*^*–/–*^ compared to wild-type animals (Fig. 5c). We also determined the cell count of CD45^+^ CD64^hi^ Ly-6C^hi^ pro-inflammatory macrophages as an additional readout for inflammation in the back skin. The number of these cells was also lower in UVB-irradiated skin of IL-1β-deficient mice as compared to wild-type animals (Fig. 5c). These results indicate that inflammatory cell recruitment into the skin in response to UVB irradiation is not only dependent on caspase-1 but additionally requires (pro)IL-1β.

### Dendritic cells and T cells, but not keratinocytes express proIL-1β in murine skin

**Fig. 6:**
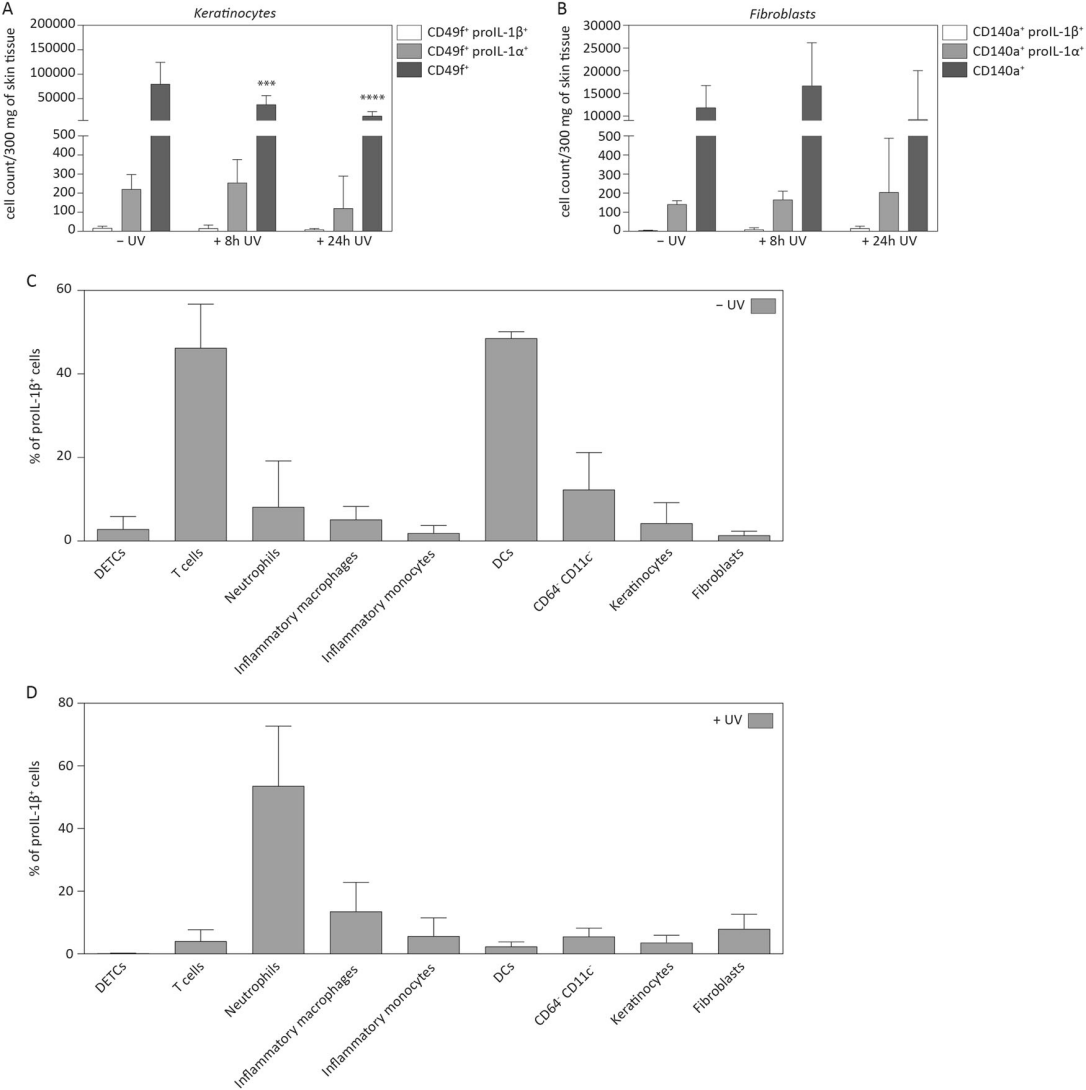
**Murine keratinocytes express only low levels of proIL-1β** **a** Numbers of keratinocytes (CD45^−^ CD49f^+^) and **b** fibroblasts (CD45^−^ CD140a^+^) expressing proIL-1α or proIL-1β in back skin of UVB-irradiated and non-irradiated wild-type mice as determined by flow cytometric analysis. The cell count of CD49f^+^ and CD140a^+^ cells per 300 mg of back skin tissue prior to and in response to UVB irradiation was quantified. Error bars represent mean ± SD of a representative experiment with *n* = 5 (8 h UV) and *n* = 10 (24 h UVB) animals/group. Two-way ANOVA with Tukey’s multiple comparison was performed, comparing non-irradiated and irradiated skin at two time points within each group (****p < 0.0001, ***p < 0.001). **c**, **d** The contribution of different cell types to the total number of proIL-1β-producing cells is indicated. Cell populations were defined as follows: CD45^+^ CD3^hi^ (DETCs), CD45^+^ CD3^+^ (T cells), CD45^+^ CD11b^+^ Ly-6G^+^ (neutrophils), CD45^+^ CD11b^+^ CD64^+^ Ly-6C^+^ (inflammatory macrophages), CD45^+^ CD11b^+^ CD64^lo^ Ly-6C^+^ (inflammatory monocytes), CD45^+^ CD11b^+^ CD64^−^ CD11c^+^ (DCs), CD45^+^ CD11b^+^ CD64^−^ CD11c^−^ (unknown cell type), CD45^−^ CD49f^+^ (keratinocytes) and CD140a^+^ (fibroblasts). **c** Wild-type mice were shaved and back skin was analysed for expression of proIL-1β in the above-mentioned cell types. **d** Wild-type mice were shaved and irradiated with UVB (300 mJ/cm^2^). Back skin of mice was harvested after 24 h and the different cell types were examined for proIL-1β expression using multicolour flow cytometry analysis. Error bars represent mean ± SD of a representative experiment with *n* = 10 animals per group. IL interleukin, DCs dendritic cells

As we were not able to detect expression of proIL-1β in murine keratinocytes in vitro, we performed flow cytometric analysis of intracellular cytokine production for proIL-1β and proIL-1α in different cell types of untreated or UV-irradiated skin. Consistent with our previous data, only very few keratinocytes that were positive for proIL-1β^+^ could be detected, and only few cells co-expressed the fibroblast marker CD140a and proIL-1β 24 h after UVB irradiation (Fig. 6a, b). However, a small percentage (around 20% of all CD45^−^ CD49f^+^/CD140a^+^ cells) of keratinocytes and fibroblasts expressed proIL-1α. These results indicate that murine keratinocytes do not produce large amounts of proIL-1β in the skin, even upon UVB irradiation. By contrast, proIL-1β was expressed by CD64^−^ CD11c^+^ dendritic cells and by CD3^+^ T cells in the skin of non-irradiated animals and by a population of CD64^−^ CD11c^−^ cells, which were not further defined (Fig. 6c). Upon UVB irradiation, most proIL-1β^+^ cells were CD45^+^ CD11b^+^ Ly-6G^+^ neutrophils, besides small numbers of CD45^+^ CD64^hi^ Ly-6C^+^ macrophages and CD45^+^ CD64^lo^ Ly-6C^hi^ inflammatory monocytes (Fig. 6d). Taken together, these results suggest that the “sunburn” reaction is entirely mediated by immune cells in murine skin, whereas keratinocytes most likely contribute to UVB-induced inflammation in human skin.

### Langerhans cells sense UVB irradiation in murine skin, but are not responsible for neutrophil recruitment

**Fig. 7:**
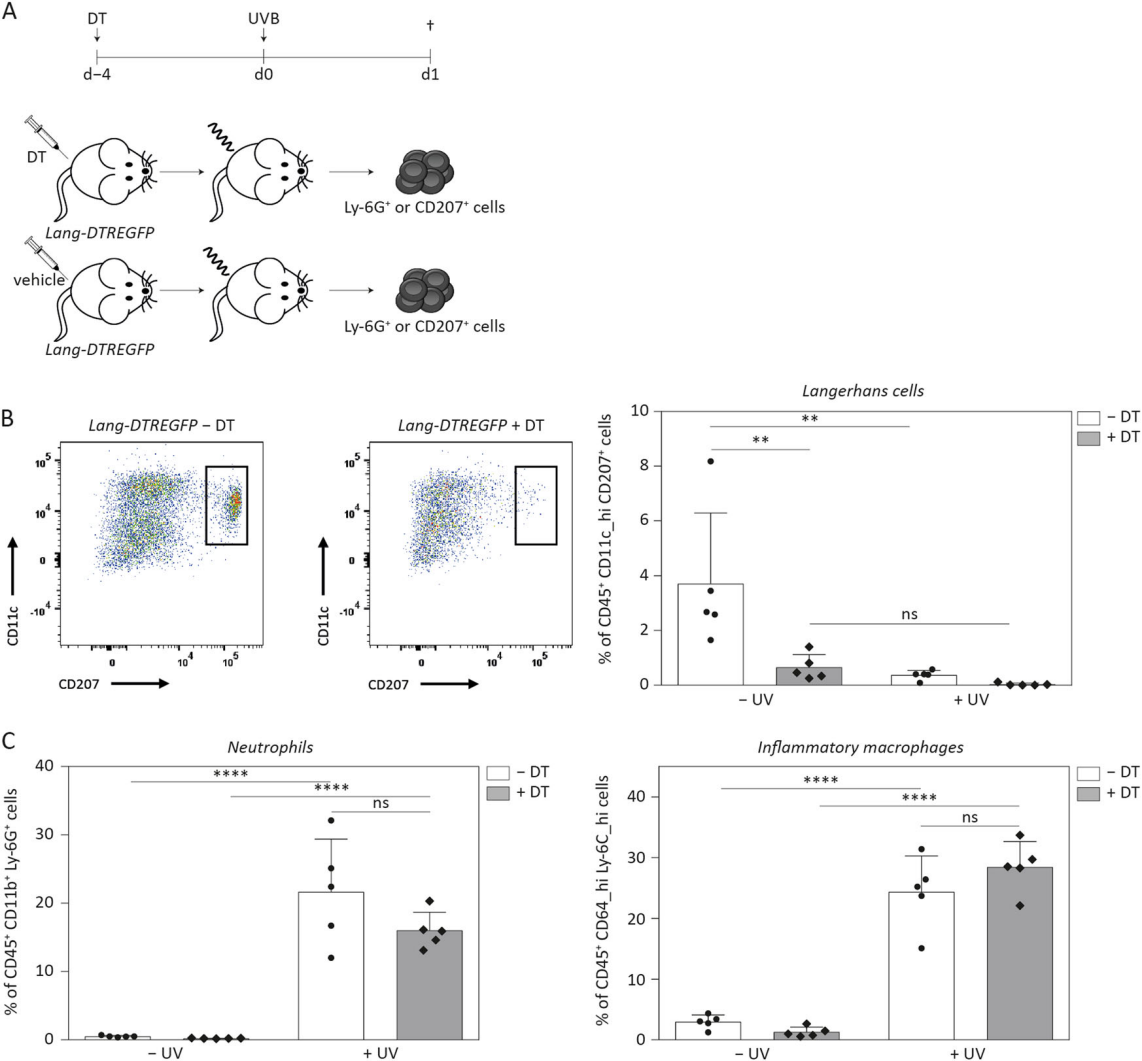
**Neutrophil recruitment upon UVB irradiation is not dependent on Langerhans cells** **a** Experimental setup of depletion of Langerhans cells in *Lang-DTREGPF* mice using diphtheria toxin (18 ng/g body weight). Control animals were injected with vehicle. After 4 days, animals were subjected to UVB irradiation (300 mJ/cm^2^), non-irradiated animals served as control. **b** Depletion of CD207^+^ Langerhans cells was controlled by multicolour flow cytometry. **c** The percentage of CD45^+^ CD11b^+^ Ly-6G^+^ neutrophils and of CD45^+^ CD64^hi^ Ly-6C^hi^ pro-inflammatory macrophages was determined by flow cytometry. Error bars represent mean ± SD of a representative experiment with *n* = 5 animals/group. One-way ANOVA with Sidak’s multiple comparison was performed (****p < 0.0001, **p < 0.01). ns not significant, DT diphtheria toxin

Our results and published data^23^ demonstrate that dendritic cells in murine skin express proIL-1β (Fig. 6c). In the epidermis, Langerhans cells (LCs) are the main type of resident dendritic cells. It has been suggested that IL-1β is necessary for LC activation and that these cells can secrete IL-1β in response to LPS stimulation^23,36^. To determine, whether LCs are important for UVB-induced caspase-1- and IL-1β-dependent inflammation, we used *Lang-DTREGFP* mice (Fig. 7a). These animals express the diphtheria toxin receptor (*DTR*) and *EGFP* downstream of the internal stop codon of the *CD207* gene, also called Langerin, which is specifically expressed in LCs. Following injection of diphtheria toxin (DT), LCs are ablated in these animals (Fig. 7b). Upon UVB irradiation of DT-treated *Lang-DTREGFP* mice and littermates injected with vehicle, we detected high levels of neutrophils as well as of pro-inflammatory macrophages in the skin. However, there was no difference between treatment groups (Fig. 7c). The number of LCs in irradiated skin of wild-type mice was strongly reduced compared to non-irradiated control mice, suggesting that UVB is sensed by LCs, which then translocate to distant lymph nodes to induce adaptive immune responses^37–39^. These results demonstrate that LCs can sense UVB, but are not responsible for caspase-1- and IL-1β-dependent neutrophil recruitment upon UVB irradiation in mice. Therefore, it seems likely that other professional immune cells such as T cells, conventional DCs or neutrophils themselves act as inducers of sunburn in mice.

## Discussion

The mouse represents one of the most important animal models for the study of mechanisms underlying human diseases, including inflammatory skin disorders and skin cancer. However, the profound differences between human and murine skin do not necessarily allow a translation of results obtained in mouse models to humans and therefore, such results need to be interpreted carefully. This is particularly critical for data obtained with UV irradiation, since mice are nocturnal animals that are rarely exposed to UVB and are moreover protected by fur. Human skin is more directly exposed to exogenous stresses and is composed of many more epidermal cell layers than murine skin^5^. Keratinocytes are the main cell type of the epidermis and implicated in the protection against endogenous and exogenous harmful stimuli^1^. In general, it is believed that murine and human keratinocytes share many common characteristics. Here we present evidence that human but not murine keratinocytes express significant amounts of proIL-1β *in vitro* and *in vivo*. Furthermore, expression of inflammasome proteins is much lower in murine keratinocytes, which provides a likely explanation for the lack of inflammasome activity in these cells.

UVB irradiation of human primary keratinocytes in culture represents a physiologically relevant model, as UVB damages keratinocytes *in vivo*, causes sunburn, and can lead to skin cancer development^7,8,40^. Most likely, sunburn in humans is caused by the activation of inflammasomes in keratinocytes, which results in pro-inflammatory cytokine secretion^8,11^. Upon sensing of diverse pathogen- or danger-associated molecular patterns, like UVB, cytosolic DNA, nigericin or nanoparticles, human keratinocytes can activate different types of inflammasomes^10,15,16,25,33^. The physiological relevance of inflammasome activation in human keratinocytes *in vitro* is supported by secretion of IL-1β and IL-18 from UVB-irradiated human skin substitutes, demonstrated in this study. Dysregulation of NLRP1 inflammasome activation can result in the development of skin disorders^18,41^, which implies an important role of inflammasome signalling in keratinocytes. It becomes increasingly evident that the NLRP1 inflammasome might be the most important inflammasome in human keratinocytes, as germline mutations in the *NLRP1* gene were identified as the cause of human skin inflammatory syndromes^18,42^. This is further supported by the identification of single nucleotide polymorphisms in *NLRP1*, which pre-disposes to skin disorders like psoriasis and vitiligo^13,43–45^. Taken together, these data indicate that inflammasome activation and subsequent secretion of IL-1β in keratinocytes of human skin is an important inflammatory pathway that can be studied *in vitro* in human primary keratinocytes.

In contrast, the results shown in this study demonstrate that murine keratinocytes do not produce and secrete detectable amounts of IL-1β, which is consistent with earlier reports^20,21^. Also in line with previous findings^21^, we detected induction of proIL-1α expression upon priming of murine keratinocytes* in vitro*. However, IL-1α was not released in response to stimuli, which induce inflammasome activation in human keratinocytes. Furthermore, IL-1α is released by murine keratinocytes upon silica dioxide treatment independently of expression of Asc^25^, suggesting that this process is inflammasome-independent. Finally, characterisation of primary murine keratinocytes in vitro revealed that besides the impaired expression of proIL-1β, stimuli, which induce inflammasome activation in human keratinocytes, are not able to cause secretion of IL-1α or IL-18. This is most likely due to very low or even undetectable expression of important inflammasome proteins.

In spite of the lack of inflammasome activation in murine keratinocytes, neutrophil influx, as well as recruitment of pro-inflammatory macrophages and monocytes were dependent on caspase-1 and proIL-1β expression, suggesting an involvement of inflammasomes in this process. At the same time, we detected expression of proIL-1β by various types of immune cells in murine skin, including T cells, dendritic cells and another, yet uncharacterized immune cell population, but not by keratinocytes.

Langerhans cells are the main antigen-presenting cell type in the epidermis and they respond to and secrete proIL-1β upon stimulation^23,46^. We indeed observed a decrease in the number of LCs upon irradiation, suggesting their activation and subsequent migration to the draining lymph nodes^38^. However, neutrophil recruitment upon irradiation was not reduced by ablation of LCs, demonstrating that LCs are dispensable for IL-1β release and subsequent neutrophil attraction and thus do not contribute to the induction of sunburn. Possible alternative candidates include other types of professional immune cells such as dendritic epidermal T cells, which are unique to murine skin, or dermal dendritic cells. In addition, it may well be that certain damage-associated small molecules released very early upon UVB irradiation, can act as chemoattractant for an initial influx of neutrophils that subsequently produce high levels of IL-1β and further promote a full-blown sunburn reaction. We do not believe that the small percentage of proIL-1β-positive keratinocytes serves as an initial trigger, as we do not have any hints from other experiments that murine keratinocytes are able to produce mature IL-1β.

In conclusion, we identified a major immunological difference between the response of human and murine skin to UVB, which may have evolved as a response to the frequent exposure of human skin to UV irradiation and the loss of the protective function of the fur in humans. Thus, it seems likely that a faster and stronger response to UV irradiation is required in humans, which is mediated by keratinocytes of the outermost layer of the skin. Future studies should address the contribution of keratinocytes vs. different immune cells to the sunburn reaction in human skin and identify the major player(s) in triggering this response in murine skin.

## Electronic supplementary material

Supplementary data

Supplementary Figure 1
