# Supplementary material for: Expression of inflammasome proteins and inflammasome activation occurs in human, but not in murine keratinocytes
Source: Cell Death Dis. 2018 Jan 18;9(2):24. doi: 10.1038/s41419-017-0009-4 (PMC5833864; doi:10.1038/s41419-017-0009-4)
Supplement: Supplementary file 1 — Supplementary data [file 41419_2017_9_MOESM1_ESM.docx]

**Supplementary data**

**Chemicals**

upLPS was purchased from Invivogen (*San Diego*, *US-CA*), IFNγ and TNFα were obtained from PeproTech (*New Jersey*, *US-NJ*), poly(dA:dT), diphtheria toxin from Sigma (*Munich, Germany*) and nigericin from Enzo Life Sciences (*New York*, *US-NY*). Release of IL-1α (eBIOSCIENCE, *San Diego, US-CA*), IL-1β (R&D Systems, *Minneapolis*, *US-MN*) and IL-18 (MBL INTERNATIONAL, *Woburn, US-MA*) was determined by ELISA, according to the instructions of the manufacturers. Cytotoxicity was quantified using the CytoTox 96 LDH assay kit according to the manufacturer’s instructions (PROMEGA, *Madison, US-WC*).

**Antibodies**

*Murine*: IL-1α (R&D systems, AF-400-NA), IL-1β (R&D systems, AF-401-NA), IL-18 (Abcam, *Cambridge, UK*; ab71495), caspase-1 (Adipogen, *San Diego*, *US-CA*; AG-20B-0042), caspase-11 (Abcam, ab180673), Asc (Adipogen, AL177), β-actin (Sigma, A5441), Nlrp3 (Alexis, *Lausen, Switzerland*; ALX-804-881-C100).

*Human*: ASC (Adipogen, AL177), CASPASE-1 (Santa Cruz, *Santa Cruz, US-CA*; sc-622), cleaved CASPASE-3 (Cell Signaling, *Danvers, US-MA*; #9661L), β-ACTIN (Sigma, A5441), IL-1β (R&D systems, MAB201), IL-18 (MBL, *Woburn, US-MA*; PM014); NLRP1 (Adipogen, AL176).

**Flow cytometry**

The following antibodies and dyes were used for the analysis of murine back skin by flow cytometry:

Table 1 Antibodies and dyes used for flow cytometry analysis

| **Antigen / Marker** | **Label / Dye** | **Antibody Clone** | | |  | **Dilution** | | **Vendor** | | **Ref. No.** | |
| --- | --- | --- | --- | --- | --- | --- | --- | --- | --- | --- | --- |
| Fixable Viability Dyes | | | | | | | | | | | |
| Live/Dead Fixable | Zombie Aqua | - | | | | | 1/500 | | BL | | 423102 |
|  | Zombie Red | - | | | | | 1/800 | | BL | | 423110 |
|  | Zombie Violet | - | | | | | 1/500 | | BL | | 423114 |
| Cell Surface Markers | | | | | | | | | | | |
| CD3ε | APC-Cy7 |  | 17A2 |  |  | 1/300 | | BL | | 100222 | |
|  | BV 785 |  | 17A2 |  |  | 1/300 | | BL | | 100231 | |
| CD4 | BV 711 |  | GK1.5 |  |  | 1/400 | | BL | | 100447 | |
|  | PE-Cy7 |  | GK1.5 |  |  | 1/400 | | BL | | 100422 | |
| CD11b | BV 650 |  | M1/70 |  |  | 1/500 | | BL | | 101239 | |
|  | BV 711 |  | M1/70 |  |  | 1/500 | | BL | | 101242 | |
| CD11c | FITC |  | HL3 |  |  | 1/500 | | BD | | 557400 | |
|  | PE-Cy5 |  | N418 |  |  | 1/500 | | BL | | 117316 | |
|  | PE-Cy7 |  | N418 |  |  | 1/500 | | BL | | 117318 | |
| CD16/CD32 | FcR block | | 93 |  |  | 1/200 | | BD | | 553142 | |
|  | PerCP-Cy5.5 |  | PC61.5 |  |  | 1/200 | | eBio | | 45-0251-82 | |
| CD45 | AF 700 |  | 30-F11 |  |  | 1/400 | | BL | | 103128 | |
|  | APC-Cy7 |  | 30-F11 |  |  | 1/400 | | BL | | 103116 | |
|  | BV 785 |  | 30-F11 |  |  | 1/400 | | BL | | 103149 | |
| CD49f | Biotin |  | GoH3 |  |  | 1/300 | | BL | | 313604 | |
|  | PE |  | GoH3 |  |  | 1/300 | | BD | | 561894 | |
| CD64 | APC |  | X54-5/7.1 |  |  | 1/200 | | BL | | 139305 | |
|  | BV 421 |  | X54-5/7.1 |  |  | 1/200 | | BL | | 139309 | |
| PDGFRα/CD140a | APC |  | APA5 |  |  | 1/200 | | BL | | 135908 | |
| CD207 (Langerin) | PE |  | B-PE |  |  | 1/300 | | BL | | 144204 | |
| F4/80 | AF 647 |  | Cl:A3-1 |  |  | 1/200 | | AbD | | MCA497A647T | |
| I-A/I-E | BV 510 |  | M5/114.15.2 |  |  | 1/1000 | | BD | | 107635 | |
| IgG1, rat | BV 421 |  | RTK2071 |  |  | acc. | | BL | | 400429 | |
|  | PE-Cy7 |  | RTK2071 |  |  | acc. | | BL | | 400415 | |
| IgG1, ms | BV 605 |  | MOPC-21 |  |  | acc. | | BL | | 400161 | |
| IgG2b, rat | PE |  | eB149/10H5 |  |  | acc. | | ebio | | 12-4031-81 | |
| Ly-6C | PerCP-Cy5.5 |  | HK1.4 |  |  | 1/400 | | BL | | 128012 | |
| Ly-6G | AF 700 |  | 1A8-Ly6g |  |  | 1/400 | | BL | | 127622 | |
|  | PerCP-Cy5.5 |  | 1A8-Ly6g |  |  | 1/400 | | BL | | 127616 | |
| TCRβ | AF 700 |  | H57-597 |  |  | 1/300 | | BL | | 109224 | |
|  | APC-Cy7 |  | H57-597 |  |  | 1/300 | | BL | | 109219 | |
| Cytokines | | | | | | | | | | | |
| IL-1α | PE |  | ALF-161 |  |  | 1/1000 | | eBio | | 12-7011-82 | |
| IL-1β Pro-form | PerCP-eFluor710 |  | C-APC |  |  | 1/1000 | | eBio | | 46-7114-80 | |
| AbD, AbD Serotec (BioRad); acc., according to control; AF, Alexa Fluor; BD, BD Biosciences; BL: BioLegend; BV, Brilliant Violet; CD, cluster of differentiation; eBio, eBioscience (Affymetrix); IL, interleukin; TCR, T cell receptor | | | | | | | | | | | |

Figure legend supplementary information

Supplementary Figure 1 Basic gating strategy and effect of UVB irradiation on the cell composition in murine back skin. (A) After exclusion of dead cells and doublets, cell types were identified using the following gating strategy: The live cell population was analysed by gating first on CD45^+^ and CD45^-^ cells, to distinguish between immune cells and non-immune cells. Among CD45^-^ cells, we distinguished CD49f^+^ keratinocytes and CD140a^+^ fibroblasts. CD45^+^ cells were classified into CD3^+^ lymphoid cells and CD11b^+^ myeloid cells. Myeloid cells were subdivided into Ly-6G^+^ neutrophils and Ly-6G^-^ cells. CD11b^+^ Ly-6G^-^ cells were further characterised as CD64^-^ CD11c^+^ dendritic cells, CD11c^-^ CD64^+^ macrophages and CD11c^-^ CD64^lo^ monocytes. (B-D) Mice were subjected to UVB irradiation using different doses (100, 300 and 500 mJ/cm^2^), which led to a dose-dependent increase in CD45^+^ CD11b^+^ Ly-6G^+^ neutrophils (B) and in CD45^+^ CD11b^+^ myeloid cells (C) and to a decrease of CD45^+^ CD3^+^ T cells (D). Error bars represent mean ± SD of a representative experiment with n=3 animals per group. One-way ANOVA with Dunnett’s multiple comparison comparing all values to those of non-irradiated mice was performed (^****^p<0.0001).
