# Supplementary figures and images for: Expression of inflammasome proteins and inflammasome activation occurs in human, but not in murine keratinocytes
Source: Cell Death Dis. 2018 Jan 18;9(2):24. doi: 10.1038/s41419-017-0009-4 (PMC5833864; doi:10.1038/s41419-017-0009-4)

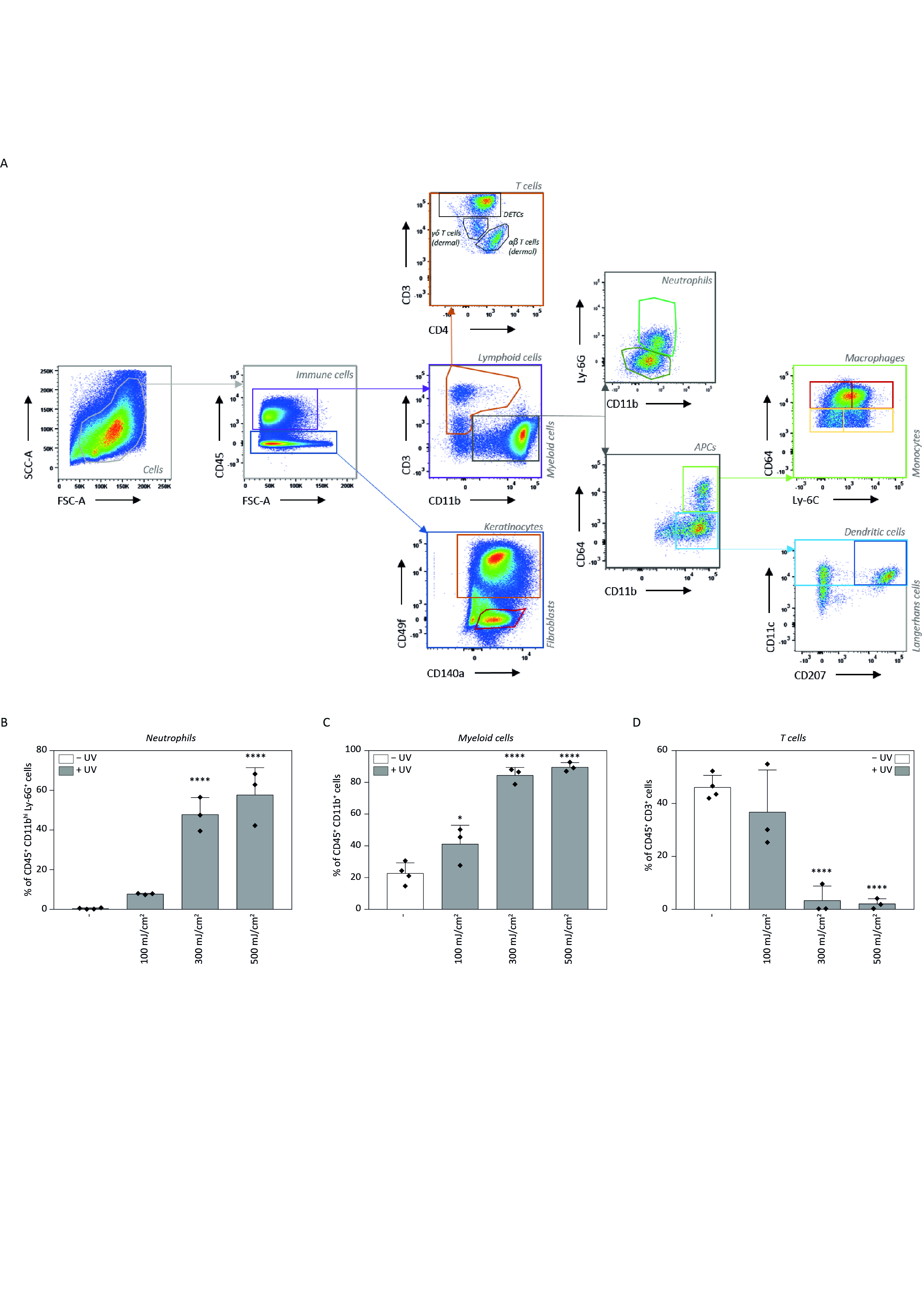

Supplement: Supplementary file 2 — Supplementary Figure 1 [file 41419_2017_9_MOESM2_ESM.tif]
